# Effects of lipid based Multiple Micronutrients Supplement on the birth outcome of underweight pre-eclamptic women: A randomized clinical trial

**DOI:** 10.12669/pjms.38.1.4396

**Published:** 2022

**Authors:** Nabila Sher Mohammad, Rubina Nazli, Hafsa Zafar, Sadia Fatima

**Affiliations:** 1 Dr. Nabila Sher Mohammad, MBBS, M.Phil Institute of Basic Medical Sciences IBMS, Khyber Medical University KMU, Peshawar, Pakistan; 2 Prof. Dr. RubinaNazli, MBBS, PGD, PhD Institute of Basic Medical Sciences IBMS, Khyber Medical University KMU, Peshawar, Pakistan; 3 Hafsa Zafar BS Nutrition, Institute of Basic Medical Sciences IBMS, Khyber Medical University KMU, Peshawar, Pakistan; 4 Dr. Sadia Fatima MBBS, PGD, PhD Institute of Basic Medical Sciences IBMS, Khyber Medical University KMU, Peshawar, Pakistan

**Keywords:** Pre-eclampsia, Lipid based Nutritional supplements, Neonatal outcome, Khyber PakhtunKhwa province Pakistan, **Trial registration:** ISRCTN15485068, April 2018:https://doi.org/10.1186/ISRCTN15485068

## Abstract

**Background and Objective:**

Maternal under nutrition and low birth weight babies are among the common tragedies of developing countries like Pakistan. Preeclampsia and its significant association with fetal growth restriction due to spiral arteries remodeling and trophoblastic invasion decreases nutritional supply to growing fetus added by maternal under nutrition. This study was designed to see the effects of lipid based nutritional supplements for pregnant and lactating women LNS-PLW on maternal and fetal outcome of pre-eclampsia.

**Methods:**

Sixty underweight pre-eclamptic women were randomly assigned into two study Groups from April 2018 to December 2019 at the antenatal units of the tertiary Health care facilities of Lady Reading Hospital, Hayatabad Medical Complex Peshawar and Civil Hospital Matta Swat, KPK Pakistan in a randomized clinical trial. Participants were on routine drugs for pre-eclampsia and Iron and Folic Acid (60mg, 400 μg) daily, while participant of Group-2 (n=30) received one sachet of Lipid based nutritional supplement for pregnant and lactating women LNS-PLW in addition daily till delivery. The birth weight, gestational age, head-circumference, and birth length of babies were measured.

**Results:**

The significant improvement found in the birth weight (p-value 0.003), gestational age (p-value 0.006), head circumference (P-value of 0.0006) and birth length (P-value of 0.0017) of babies of Group-2 women. We observed that addition of Lipid based nutritional supplement for pregnant and lactating women LNS-LPW improved the birth outcome in underweight women of pre-eclampsia.

**Conclusion:**

The Prenatal supplementation of Lipid based nutritional supplement for pregnant and lactating women LNS-PLW can improve birth weight, gestational age, length and head circumference of babies of underweight preeclamptic women.

## INTRODUCTION

Preeclampsia is a multisystem disorder (proteinuria, hypertension and edema after 20 weeks of gestation) originating from placenta. Although an immunological deficiency is suggested, the condition is unusual in having no known cause. In blood relatives, the incidences are significantly higher (mothers, daughters, sisters, and granddaughters).^1^ Pregnancy-induced hypertension diseases, including preeclampsia, have been shown to occur more frequently in first pregnancies and less frequently in subsequent pregnancies.^2^

It is responsible for several short and long term maternal and neonatal complications. Preeclampsia is the second most common cause of maternal mortality in the world responsible for 10-15% of maternal death.^3^ It affects 3-5% of pregnant women worldwide and reported one of the main causes of maternal mortality, morbidity and still births.^4^ The prevalence of complication with maternal and fetal outcome is 19% with pre-eclampsia in Pakistan.^5^ Pakistan is the world’s third country having highest newborn deaths per year (194 000 deaths in 2010). 5 According to recent ranking Pakistan is third in terms of burden of fetal, maternal and child mortality.^3^ Our country has poorest pregnancy outcomes worldwide, maternal mortality was found to be 319 per 100,000 live births.^6^

The incidence of pregnancy induced hypertension including pre-eclampsia and eclampsia in Sukkar Pakistan was 5.56%.^7^ The neonates of pre-eclamptic woman are affected due to vasoconstriction of spiral uterine arteriole decreasing blood supply to growing baby in the uterus. Hypertensive disorders are correlated with poorer perinatal outcomes such as preterm birth, intrauterine growth retardation, Low birth weight and several other fetal complications.^8^

In underdeveloped countries, malnutrition and anaemia during pregnancy are two of the most common causes of maternal morbidity and mortality, both of which are associated with fetal outcome.^3^,^9^,^10^ The nutritional status during pregnancy, maternal anemia, pregnancy induced hypertension and pre-eclampsia plays important role in fetal development and growth.^11^ Pregnant women are at higher risk of deficiency of macro and micronutrients, due to added burden of the growing placenta, fetus, and maternal metabolism.^11^

Unfortunately, under nourished pregnant women and the incidence of pre-eclampsia is high in our country. Studies have reported positive association of multiple micronutrients deficiency and development of pre-eclampsia.^7^ Lipid base nutritional supplements for pregnant and lactating women are used in under developed countries to improve the nutritional status of pregnant and lactating women and have positive impact on pregnancy outcomes.^7^

However, as per our knowledge no research has been conducted to observe effects of Lipid based nutritional supplement for pregnant and lactating women LNS-PLW on underweight pre-eclamptic women. Keeping in mind the positive impact of lipid based nutritional supplement for pregnant and lactating women on the nutritional status of normotensive pregnant women and improved neonatal and children growth and development outcome we designed this study.^12^-^14^

## METHODS

In this, randomized clinical trial 60 underweight pre-eclamptic nonsmoker^15^ primigravida aged between 15-35years were included from April 2018 to December 2019 at the antenatal units of the tertiary Health care facilities of Lady Reading Hospital, Hayatabad Medical Complex Peshawar and Civil Hospital Matta Swat, Khyber Pakhtunkhwa province of Pakistan. Underweight primigravida, with sign and symptoms of pre-eclampsia i.e. hypertension, proteinuria and edema.

### Exclusion Criteria:

History of hypertension, essential hypertension, Diabetes Mellitus, renal and liver disorders. Those women who were allergic to supplements were excluded.

### Ethical approval:

This trail was approved by the Advanced Study and Research Board of KMU (ASRB Approval No: DIR/KMU-AS&RB/EN/000527) on 18^th^ August 2016 and got approved by the institutional ethical committee (DIR/KMU=EB/EN/000314) on 27^th^ October 2016.

**Fig.1:**
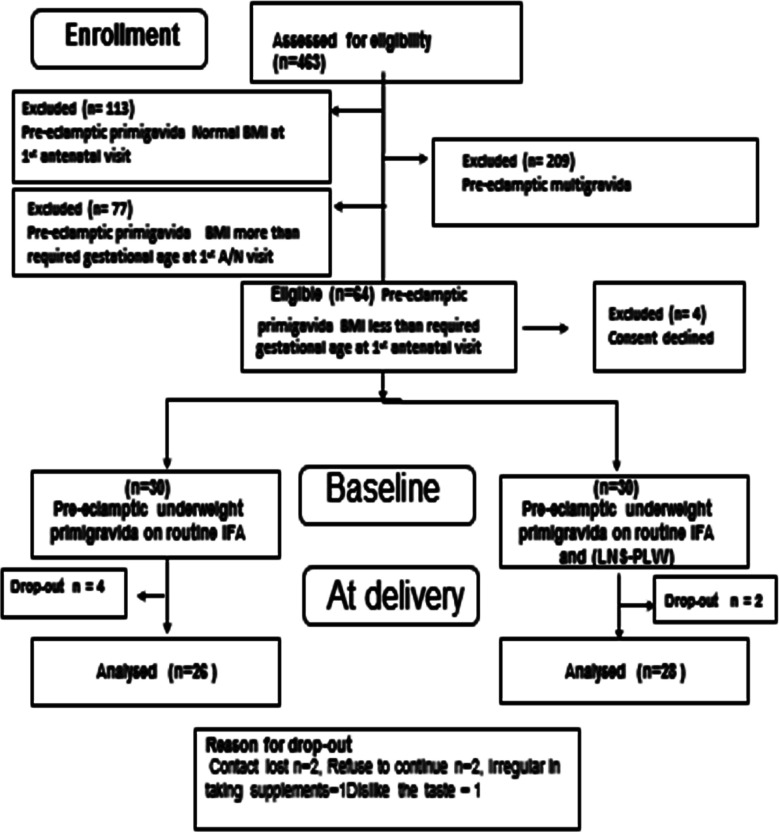
Flowchart diagram of sample selection.

A total of 60 underweight pre-eclamptic primigravida nonsmokers were recruited after screening 463 pre-eclamptic women Fig.1. Eligible Basal Metabolic Rate less than required BMI of gestational age were recruited by taking written informed consent. Participants were randomly assigned in two Groups by using computer randomizer (version 3.0). All participants received conventional treatment of pre-eclampsia and Iron and folic acid IFA (60mg Iron, 400ug Folic Acid) daily. In addition, participants in Group-2 received a 75gm sachet of Lipid based nutritional supplement for pregnant and lactating women LNS-PLW daily till delivery. To our knowledge, this study is novel which is first to report the effect of Lipid based nutritional supplement for pregnant and lactating women in underweight pre-eclamptic women in their first pregnancy. The participant compliance was good due to their cooperation and main researcher contact and follow-up.

Socio-demographic data, personal and family history of pregnancy induced hypertension was recorded. The age, anthropometric measurements, gestational age, height, weight, BMI, systolic and diastolic blood pressure and proteinuria were measured.

LNS-PLW is the World Food Program supplements packed in 75 grams ready to use sachet, especially designed for pregnant and lactation women for once-a-day use (Table-I).^12^,^16^-^18^ The main researcher herself provided Lipid based nutritional supplement for pregnant and lactating women twice a month and after fifteen days the empty sachets were collected back in order to check the compliance. Any leftover in the sachet were also calculated by the main researcher. Apart from that the main researcher telephonically called each participant on alternate days to remind them to take their medication and LNS-PLW.

**Table-I:** Composition and nutritional value of nutritional supplement LNS-PLW.

Total Energy	400kcal		

Nutrients	value	Nutrients	value
Protein	10.5g	Vit B2	1.57mg
fat	24g	Vit B3	9.75mg
Thiamin	0.75mg	B5	3.0mg
Sugars	1g	B6	1.35mg
Retinol (Vit A)	412mcg	B7	45mcg
Folates(Vit B9)	247mcg	Vit E	12mg
Cholecalciferiol (Vit D)	11.2mcg	B12	2.0mcg
Calcium (Ca)	400mg	Vit C	45mg
Copper (Cu)	1.0mg	Vit K	20.2mcg
Iron (Fe)	7.5mg	Iodine	75mcg
Magnesium (Mg)	112mg	Selenium (Se)	15mcg
Manganese (Mn)	0.9mg	Sodium (Na)	mg
Phosphorus (P)	337mg	Zinc (Zn)	8.2mg
Potassium (K)	675mg		

The height and weight of participants were measured by following specific protocols. For measurement of height and weight portable stadiometer - Seca Leicester 214 and Beurer digital glass weight scale-GS 200 Allium were used. The blood pressure of patients was measured three times with 10 minutes interval with and then mean was measured. Protein in urine was measured by dipstick method by main researcher. The mode of delivery of participants (normal vaginal delivery, caesarean section) was also recorded.

The primary birth outcomes (birth weight, length) and secondary outcomes (gestational age at delivery, head circumference), were measured. WHO, worldwide Child Growth Standards were used to determine birth weight (Seca334), length by portable infantometer (Seca417) and head circumference (Seca212 tape) of study Groups babies and defined preterm delivery as delivery before 37 weeks of gestation, low birth weight as a birth weight < 2500 g.^19^

The socio demographic risks for low birth weight in study Groups like age of mothers, education, income, living status, BMI at time of 1^st^ antenatal visits and at time of enrolment, antenatal visits and consumption of Iron, Folic acid and lipid based nutritional supplement for pregnant and lactating women were recorded for analysis and comparison.

### Statistical analysis:

The patient’s demographic data was analyzed as descriptive statistics and was presented as mean ± standard deviation. The normality of data was tested by Shapiro-Wilk. The baseline data of anthropometric was compared between Groups by un-paired sample t-test. All analyses were performed using SPSS software, version 22 “SPSS (IBM, Armonk, NY, USA). P<0.05 was considered significant.

## RESULTS

In this randomized clinical trial sixty-four underweight pre-eclamptic nonsmokers primigravida were found eligible, four of them declined consent and sixty gave consent and started the trial (Fig.1).

### Socio demographic characteristics:

There was no difference in socio demographic characteristic of both Groups, lack of education and poor financial status was common in both study Groups (Table-II).

**Table-II:** Socio demographic characteristics of study Groups.

Parameters	Control IFA Group-1 (n=26)	Interventional LNS-PLW Group-2 (n=28)
***Age (years)***		
15-19	9	6
20-24	11	10
25-29	6	12
***Participant education***		
No education	20	22
Primary	-	2
Middle	1	2
Matric	2	1
High secondary	2	-
Graduate	-	1
***Participant occupation***		
Housewife	25	28
Working women	1	-
***Husband education***		
No education	13	10
Primary	1	-
Middle	-	4
Matric	6	7
High secondary	1	-
Graduate	4	6
***Husband occupation***		
Laborer	16	18
Businessman	7	5
Teacher	2	2
Engineer	-	1
Govt Job	1	-
***Family income (Rs)***		
<15000/-	14	12
15000/- -25000/-	2	5
26001/- -35000/-	2	2
36001/- -45000/-	2	2
>45000/-	6	7
***Family history of preeclampsia/PIH***		
Nil	14	18
Mother	8	8
Sister	4	2
***Antenatal visits***		
2-3	19	7
4-5	7	21
***House status***		
Pakka	12	15
Kacha/ Semi pakka	14	13
***Pregnancy registered***		
Yes	26	28
No	-	-
***Sex of baby***		
Boy	14	15
Girl	12	13
***Family History of hypertenssion***	8	10
Mother	5	8
Father	3	2

The base line obstetrics and clinical characteristics were similar in both Groups i.e. Iron and Folic acid Group-1 and lipid based nutritional supplement for pregnant and lactating women Group-2. No significant difference was found in the mean age of both Groups (Group-1 = 21.76± 3.33;Group-2 = 23.14 ± 3.54,P = 0.149).Similarly no significant difference was detected in Basal Metabolic Rateand gestational age at time of enrollment i.e. BMI (Group-1 = 27.99±2.68 kg/m^2^; Group-2 = 26.21 ± 3.39kg/m^2^, P= of 0.038) and gestational age (Group-1 = 30.30 ± 1.93weeks ; Group-2 = 29.96±2.00 weeks, P= 0.525) Table- III.

**Table- III:** Clinical and obstetric characteristics of study Groups.

Parameters	Control IFA Group-1 (n=26) Mean ± SD	Interventional LNS-PLWGroup-2 (n=28) Mean ± SD	P-value
Age (years)	21.76 ± 3.33	23.14 ± 3.54	0.149
BMI at 1^st^ visitKg/m^2^	19.61±1.33	19.67±0.85	0.844
BMI at enrollment kg/m^2^	27.99 ± 2.68	26.21 ± 3.39	0.038
BMI at delivery kg/m^2^	27.12 ± 1.40	27.24 ± 1.03	0.729
Gestational age at 1^st^ visit from record (weeks)	19.84±3.5	20.5±3.14	0.450
Gestational age at enrolment (weeks)	30.30 ± 1.93	29.96 ± 2.00	0.525
Gestational age at delivery (weeks)	36.88 ± 1.55	38.64 ± 0.78	o.006
Systolic blood pressure at enrollment (mmHg)	146.53 ± 9.35	145 ± 6.93	0.493
Diastolic blood pressure at enrollment(mmHg)	96.15 ± 5.34	95.53 ± 4.37	0.643
Proteinuria at enrolment	1.892 ± 0.737	1.884 ± 0.765	0.968
Supplements Consumed (weeks)	6.82 ± 1.89	8.76 ± 1.96	0.0005

There was no significant difference between systolic, diastolic blood pressure and levels of protein in the urine at enrollment in both Groups i.e. systolic blood pressure (Group-1 = 146.53±9.35mmHg; Group-2= 145±6.93mmHg, P = 0.493), diastolic blood pressure (Group-1 = 96.15±5.34mmHg; Group-2 = 95.53±4.37mmHg, P =0.643) and protein in urine (Group-1 = 1.892±0.737; Group-2 = 1.884±0.765, P= 0.968) Table- III.

### Obstetric and clinical characteristics at delivery:

The gestational age at delivery in Group-2 was significantly more as compared toGroup-1 i.e. (Group-1= 36.88±1.55weeks; Group-2= 38.64±0.78 weeks= 0.006) but with no significant change in their BMI at delivery i.e. (Group-1= (27.12±1.40 kg/m^2^; Group-2=27.24±1.03 kg/m^2^, P= 0.729). The participants of Group-2 consumed lipid based nutritional supplement for pregnant and lactating woman-PLW in addition to Iron and Folic acids IFA about 8.76±1.96 weeks. Table- III.

### Pregnancy and fetal outcomes:

The birth outcomes of study are summarized in Table-IV. The number of normal vaginal deliveries are more in Group-2 (Group-1 = 18(69%); Group-2 = 22(78%)) as compared to Group-1. While the number of cesarean section are more in Group-1as compared to Group-2 i.e. Group-1=8 (31%); Group-2= 6 (21%).One stillbirth and 1 intrauterine death (IUD) occurred in Group-2 while two intrauterine death IUD, 9 preterm (<37weeks) babies were born in Group-1.

**Table-IV:** Gestational age, birth weight, height, and head circumference of new born babies of study Groups.

Parameters	Control Group-1 (n=26) Mean ± SD	Interventional Group-2 (n=28) Mean ± SD	P-value
Gestational age at birth (weeks)	36.88 ± 1.55	38.64 ± 0.78	0.006
Birth weight (kgs)	2.369 ± 0.205	2.49 ± 0.083	0.0039
Birth Length (cms)	46.97 ± 0.737	47.54 ± 0.53	0.0017
Head circumference (cms)	31.29 ± 0.42	31.68 ± 0.36	0.0006

There was significant improvement in the birth weight, Length and head circumference of lipid based nutritional supplement for pregnant and lactating womenLNS-PLWGroup-2 babies as compared to Iron and Folic acid IFA Group-1 i.e. birth weight (Group-1 = 2.369 ± 0.205kgs; Group-2= 2.49±0.083kgs, P=0.0039),length(Group-1 = 46.97 ± 0.737cms; Group-2= 47.54± 0.53cms, P=0.0017) and head circumference (Group-1 = 31.29 ± 0.42 comes ; Group-2=31.68± 0.36cms,P= 0.0006).Table- IV.

Less number of LBW babies delivered in lipid based nutritional supplement for pregnant and lactating women LNS-PLW Group-2 as compared to Iron and Folic acid IFA Group-1 i.e. Group-1 = 61% (16/26); Group-2=29% (8/28).Table-V.

**Table- V:** Birth outcome of study Groups.

Parameters	Control Group-1 (n=26)	(%)	Interventional Group-2 (n=28)	(%)
Baby boy	14	53.84%	15	53.57%
Baby girl	12	46.15%	13	46.43%
Preterm / premature (<37 weeks)	9	34.6%	-	-
Low birth weight (<2.5kgs)	16	61.5%	8	28.5%
Stillbirth	-	-	1	3.5%
Intrauterine death (IUD)	2	7.69%	1	3.5%
Mode of delivery				
NVD	18	69.3%	22	78.5%
C- Section	8	30.7%	6	21.5%

## DISCUSSION

Hypertensive disorders of pregnancy are associated with maternal and fetal mortality and morbidity.^2^, ^20^ Maternal malnourishment during pregnancy also impact growing fetus and increases the risk of low birth weight, small for gestational age, intrauterine growth retardation, stillbirth, preterm birth and neonatal mortality.^21^

The current study was designed with the aim to determine the effect of lipid base multiple micronutrient nutritional supplement for pregnant and lactating women LNS-PLW on neonatal outcomes of underweight pre-eclamptic women in their first pregnancy.

In this nutritional trial, the supplementation of LNS-PLW during antenatal periods of pre-eclamptic nonsmoker underweight women showed positive impact on fetal outcome of interventional Group. Similarly, other studies have also reported positive impact of using lipid based nutritional supplements on nutritional status of pregnant women and improved neonatal and children growth and development.^17^,^22^

In current study the low birth weight LBW babies delivered in LNS-PLW Group-2 were 29% as compared to Iron and Folic acid IFA Group-1 which was 61%. This decline in the percentage of low birth weight babies in Group-2 might be attributed to the provision of supplement as it provide 400 kcal extra energy per day over and above the habitual energy intake. Other studies have also reported high percentage of low birth weight in pre-eclamptic women. In a study conducted in Sukkur Pakistan 50% of pre-eclamptic women of study population, gave birth to low birth weight babies.^7^ The percentage of low birth weight babies as a whole in our study population was 45%, this improvement may confirm the effectiveness of lipid based nutritional supplement for pregnant and lactating women LNS-PLW on birth weight. In a study conducted on primiparous women of Ghana reported low birth weight babies in lipid based nutritional supplement Group (27/307)9% vs Iron and Folic acid (44/305)15%.^16^ In our study the percentage of low birth weight babies was more in comparison to Ghana study because the duration of supplementation in our study was only 20 weeks, however in Ghana study the supplements were provided throughout the antenatal period. Other studies have also documented effectiveness of multiple micronutrient supplements among anemic women which reduces the risk of low birth weight to 19% in comparison to Iron and Folic acids IFA Group.^16^,^22^,^23^

Current study demonstrated that the use of LNS-PLW improved significantly the birth weight of the babies in Group-2 in comparison to IFAGroup-1.Our findings are similar to the findings of other studies in which the use of lipid based nutritional supplements during antenatal periods significantly improved the birth weight of babies.^18^,^24^ However, few of the studies documented no improvement in the birth weight of the babies by the use of lipid based supplements during antenatal period. Reason might be that the nutrient absorption is affected when consumed with food rather than fasting intake.^25^,^26^

In our study the gestational age of the babies in the LNS-PLW Group-2 at birth improved significantly than IFA Group-1. Litrature has documented controversial findings on the improvement of the gestational age with the use of supplements. Some reported improvement in the gestational age.^18^,^27^ While other found no effect of supplementation on gestational age.^16^ Current finding of our study is similar to the findings of other studies in which the gestational age at birth improved with lipid based nutritional supplement for pregnant and lactating women supplementation.^18^,^27^ However, on the other hand in a study supplementation of lipid based supplement had no significant effect on the gestational age at delivery.^16^ No preterm delivery occurred in the supplemented Group-2 of our study while (9/26) 34% of preterm babies delivered to Group-1 women. The incidence of preterm deliveries in our Group-1 is more as compared to the other studies on preeclamptic women which reported the incidence of 25%.^7^ This increase in the percentage of preterm deliveries might be due to the reason that our participants are underweight pre-eclamptic.

The birth length and head circumference were also improved in LNS-PLW Group-2 in current study. These findings of our study are similar to the finding by other similar studies in which supplementation improved the birth length^9^ and the head circumference in the interventional Groups.^18^ However, the Ghana study reported no improvement in birth length of newborn babies in supplement consumption Group.^16^ They claimed that taking various micronutrient supplements without adding macronutrients was insufficient to increase birth size.

When the mode of delivery was compared in this study, 78% women of LNS-PLW Group-2 delivered normal vaginally while 31% in IFA Group-1women were delivered by cesarean section. In the study of rural Bangladesh the use of supplementation had no effect on the number of cesarean deliveries in lipid based supplements consumption Group.^14^

### Limitations of the study:

This study was conducted in only two areas of KPK in which only few participants were recruited due to insufficient resources and funds. Therefore, we recommend a multicentre trial, involving more participants from different areas and with the longer duration of supplementation. Due to cultural constraint husbands were not accompanied during their antenatal visits so their anthropometric biodata could not record.

## CONCLUSIONS

We conclude that antenatal supplementation of lipid based nutritional supplement for pregnant and lactating women LNS-PLW to underweight pre-eclamptic women in the province of Khyber PakhtunKhwa (KPK), Pakistan improved birth outcomes. Pre-eclamptic women are at higher risk for fetal growth restriction, LNS-PLW increase birth weight, length, head circumference and gestational age at delivery. Therefore, it can be recommended along with Iron and Folic acids to pre-eclamptic women to reduce maternal and fetal morbidity and mortality.

## References

[1] Cooper D.W, Brennecke S.P, Wilton A. N (1993). Genetics of pre-eclampsia. Hypertens Pregnancy.

[2] Robillard PY, Dekker GA, Hulsey TC (1999). Revisiting the epidemiological standard of preeclampsia:primigravidity or primipaternity?. Eur J Obstet Gynecol Reprod Biol.

[3] Nkamba DM, Vangu R, Elongi M, Magee LA, Wembodinga G, Bernard P (2020). Health facility readiness and provider knowledge as correlates of adequate diagnosis and management of pre-eclampsia in Kinshasa, Democratic Republic of Congo. BMC Health Serv Res.

[4] Davis EF, Newton L, Lewandowski AJ, Lazdam M, Kelly BA, Kyriakou T (2012). Pre-eclampsia and offspring cardiovascular health:mechanistic insights from experimental studies. Clin. Sci. (Lond.).

[5] Aziz R, Mahboob T (2007). Pre-eclampsia and lipid profile. Pak J Med Sci.

[6] Aziz A, Saleem S, Nolen TL, Pradhan NA, McClure EM, Jessani S (2020). Why are the Pakistani maternal, fetal and newborn outcomes so poor compared to other low and middle-income countries?. Reproductive Health.

[7] Un Nisa S, Shaikh A.A, Kumar R (2019). Maternal and Fetal Outcomes of Pregnancy-related Hypertensive Disorders in a Tertiary Care Hospital in Sukkur, Pakistan. Cureus.

[8] Palei A.C, Spradley F.T, Warrington J. P, George E. M, Granger J. P (2013). Pathophysiology of hypertension in pre-eclampsia:a lesson in integrative physiology. Acta Physiologica.

[9] Lassi ZS, Padhani ZA, Rabbani A, Rind F, Salam RA, Das JK (2020). Impact of Dietary Interventions during Pregnancy on Maternal, Neonatal, and Child Outcomes in Low- and Middle-Income Countries. Nutrients.

[10] FW Lone, RN Qureshi, F Emmanuel (2004). Maternal anaemia and its impact on perinatal outcome in a tertiary care hospital in Pakistan. Eastern Mediterr Health J.

[11] Muthayya S (2009). Maternal nutrition &low birth weight-what is really important. Indian J Med Res.

[12] Das JK, Hoodbhoy Z, Salam RA, Bhutta AZ, Valenzuela-Rubio NG, Prinzo Z (2018). Lipid-based nutrient supplements for maternal, birth, and infant developmental outcomes. Cochrane Database of Syst Rev.

[13] Ali F (2014). Assesment of dietary diversity and nutritional status of pregnant women in Islamabad Pakistan. JAMC.

[14] Mridha MK, Matias SL, Paul RR, Hussain S, Sarker M, Hossain M (2017). Prenatal Lipid-Based Nutrient Supplements Do Not Affect Pregnancy or Childbirth Complications or Cesarean Delivery in Bangladesh:A Cluster-Randomized Controlled Effectiveness Trial. J Nutr.

[15] Dekker G, Sibai B (2001). Primary, secondary, and tertiary prevention of pre-eclampsia. Lancet.

[16] Adu-Afarwuah S, Lartey A, Okronipa H, Ashorn P, Zeilani M, Peerson JM (2015). Lipid-based nutrient supplement increases the birth size of infants of primiparous women in Ghana. Am J Clin Nutr.

[17] Haider BA, Bhutta ZA (2017). Multiple-micronutrient supplementation for women during pregnancy. Cochrane Database Syst Rev.

[18] Mridha MK, Matias SL, Chaparro CM, Paul RR, Hussain S, Vosti SA (2016). Lipid-based nutrient supplements for pregnant women reduce newborn stunting in a cluster-randomized controlled effectiveness trial in Bangladesh. Am J Clin Nutr.

[19] De Onis, M Onyango A, Borghi E, Siyam A, Blossner M, Lutter C (2012). Worldwide implementation of the WHO Child Growth Standards. Public Health Nutr.

[20] Christian P (2010). Micronutrients, birth weight, and survival. Annu Rev Nutr.

[21] Badshah S, Mason L, McKelvie K, Payne R, Lisboa PJ (2008). Risk factors for low birthweight in the public-hospitals at Peshawar, NWFP-Pakistan. BMC Public Health.

[22] Bourassa MW, Osendarp SJM, Adu-Afarwuah S, Ahmed S, Ajello C, Bergeron G (2019). Review of the evidence regarding the use of antenatal multiple micronutrient supplementation in low- and middle-income countries. Ann NY Acad Sci.

[23] Shah PS, Ohlsson A, Birth W, Preterm B, Knowledge Synthesis Group on Determinants of Low (2009). Effects of prenatal multimicronutrient supplementation on pregnancy outcomes:a meta-analysis. CMAJ.

[24] West KP, Shamim AA, Mehra S, Labrique AB, Ali H, Shaikh S (2014). Effect of maternal multiple micronutrient vs iron-folic acid supplementation on infant mortality and adverse birth outcomes in rural Bangladesh:the JiVitA-3 randomized trial. JAMA.

[25] Dwarkanath P, Hsu J.W, Tang G.J, Anand P, Thomas T, Thomas A (2016). Energy and Protein Supplementation Does Not Affect Protein and Amino Acid Kinetics or Pregnancy Outcomes in Underweight Indian Women. J Nutr.

[26] Nga HT, Quyen PN, Chaffee BW, Diep Anh NT, Ngu T, King JC (2020). Effect of a nutrient-rich, food-based supplement given to rural Vietnamese mothers prior to and/or during pregnancy on birth outcomes:A randomized controlled trial. PLoS One.

[27] Barton J.R, O'Brien J, M, Bergauer N.K, Jacques D. L, Sibai B. M (2001). Mild gestational hypertension remote from term:progression and outcome. Am J Obstet Gynecol.

